# Quality of life following medial patellofemoral ligament reconstruction combined with medial tibial tubercle transfer in patients with recurrent patellar dislocation: a retrospective comparative study

**DOI:** 10.1186/s13018-022-03310-2

**Published:** 2022-09-14

**Authors:** Kuo Hao, Ao Feng, Lingce Kong, Fei Wang

**Affiliations:** Department of Orthopaedic Surgery, Third Hospital of Hebei Medical University, Shijiazhuang, 050051 Hebei China

**Keywords:** Medial patellofemoral ligament reconstruction, Tibial tubercle transfer, Quality of life, Patellar dislocation

## Abstract

**Background:**

Because the patients undergoing medial patellofemoral ligament reconstruction (MPFLr) combined with medial tibial tubercle transfer (TTT) procedure are usually young and active, the quality of life (QoL) is also an important prognostic factor for patients with recurrent patellar dislocation. Assessing QoL can provide more useful and accurate evidence for the effects of this procedure. This study aimed to evaluate QoL following MPFLr combined with TTT, compared with isolated MPFLr (iMPFLr).

**Methods:**

Fifty-one patients who underwent iMPFLr + TTT and 48 patients who underwent iMPFLr were included. Clinical evaluation included QoL (EQ-5D-5L and EQ-5D VAS), functional outcomes (Kujala, Lysholm and Tegner activity scores), physical examinations (patellar apprehension test and range of motion) and redislocation rates. Radiological evaluation included patellar tilt angle and bisect offset. These preoperative and postoperative results were compared between groups at baseline and the final follow-up. The paired and independent *t* tests were used for the data following a normal distribution. Otherwise, the Wilcoxon and Mann–Whitney *U* tests were used to analyze the differences. Categorical variables were compared by chi-square or Fisher’s exact test.

**Results:**

All of the QoL (EQ-5D-5L and EQ-5D VAS), clinical results and radiological outcomes significantly improved in both groups at the final follow-up, with no significant differences between groups. There was no significant difference in five dimensions of EQ-5D at the final follow-up, although percentages of people with problems of mobility and pain/discomfort were higher in the MPFLr + TTT group. Female patients had lower EQ-5D index and EQ-5D VAS compared with male patients in both groups at the final follow-up, but there was only a significant difference in the EQ-5D VAS.

**Conclusions:**

Both MPFLr + TTT and iMPFLr groups obtained similar and satisfactory improvements in the QoL, clinical results and radiological outcomes, indicating that MPFLr combined with TTT is a safe and effective procedure, which can significantly improve the QoL for patients with recurrent patellar dislocation in cases of pathologically lateralized TT. However, female patients obtained lower QoL than males.

## Background

Recurrent patellar dislocation (RPD) is the most common complication of acute patellar dislocation, which is one of the important causes of disability in children and young adolescents, with an annual incidence up to 147.7/100,000 among patients aged 14 to 18 years [1–4]. Patellar stability depends on the bony structure of patella and femoral trochlea, restraint of soft tissues, lower limb alignment and coordination of muscles around the knee [5]. The etiology of RPD is multifactorial, including medial patellofemoral ligament (MPFL) injuries, abnormally lateralized tibial tubercle (TT), femoral trochlear dysplasia, patella alta, Wiberg type C patella, increased femoral anteversion and genu valgum [6–10].

The MPFL is the primary static soft tissue restraint which restrains against lateral subluxation and dislocation of the patella, especially between 0° and 30° of knee flexion [9]. Therefore, injury or deficiency of MPFL is one of the predisposing factors for RPD. In most cases, an acute traumatic patellar dislocation leads to MPFL rupture [8, 11]. Therefore, an anatomical repair MPFL is necessary to prevent redislocation of patella [11]. Although various procedures have been reported for the treatment of RPD, MPFL reconstruction (MPFLr), which is one of the proximal realignment surgical techniques, has become an increasingly common and popular procedure in the last decade, with satisfactory clinical outcomes, reduced redislocation rates and improved quality of life (QoL) although surgical techniques and graft types are various[5, 8, 9, 12–14]. However, it is necessary to consider the potential risk factors before isolated MPFLr (iMPFLr) [15–17].

The lateralized TT, which can be measured by TT–trochlear groove (TT–TG) distance, leads to the increase in Q angle and a lateral force on the patella, thus damaging the normal patellar tracking [8, 18]. The iMPFLr could not achieve promising results due to increased graft tension and potential failure caused by the TT lateralization, which produces anisometry in MPFLr [19]. Therefore, a combination of MPFLr and TT transfer (TTT) for patients with an increased TT–TG distance, especially when TT–TG distance is greater than 20 mm, should be taken into consideration, with the purpose of addressing both patellar dislocation and patellar maltracking at the same time to restore the optimal position of patella relative to the femoral trochlea [5, 18, 20]. A systematic review showed that MPFLr combined with TTT is a safe and effective surgery, with a low to moderate risk of complications and overall good results [21]. However, flexion deficits, strength deficiencies, a slower recovery process and a prolonged return to sport time after MPFLr with TTT compared with iMPFLr were reported [12, 22]. In addition, the additional TTT increases the operative time and the risk of tibial fracture and reoperation because of symptomatic hardware removal [23, 24]. Overcorrection of TT–TG distance can lead to medial cartilage wear and instability, thus promoting medial osteoarthritis [25].

Because the patients undergoing MPFLr with TTT procedure are usually young and active, the QoL is also an important prognostic factor for RPD. Assessing postoperative QoL, with clinical and radiological outcomes, can further provide more useful and accurate evidence for the beneficial effects of this procedure. However, to our knowledge, no study has evaluated the QoL following combined MPFLr and TTT for RPD. The five-level EuroQol five-dimensional questionnaire (EQ-5D-5L) is a generic and standardized instrument for describing and valuing health-related QoL on five dimensions of health: mobility, self-care, usual activities, pain/discomfort and anxiety/depression [26, 27]. The visual analogue scale (EQ-VAS) was also used in this study [26]. EQ-5D-5L has increased reliability and sensitivity and decreased ceiling effects compared with previous EQ-5D-3L [28].

The main aim of the present study was to evaluate postoperative QoL, clinical and radiological outcomes following combined MPFLr and TTT, compared with iMPFLr in patients with RPD in cases of pathologically lateralized TT. It was hypothesized that combined MPFLr and TTT would significantly improve both QoL and other results for RPD.

## Methods

### Patient selection

This retrospective study was approved by the ethics committee of the Third Hospital of Hebei Medical University and informed consent was obtained from the patients. All patients of RPD who underwent MPFLr from January 2017 to April 2020 were reviewed. The inclusion criteria were: (1) two or more episodes of patellar dislocation; (2) a history of recurrent patellar instability with symptoms of patellar instability and positive apprehension sign; (3) lateral subluxation or dislocation of the patella through computed tomography (CT) or magnetic resonance (MR) images; (4) skeletal maturity. The exclusion criteria were: (1) previous trochleoplasty or TTT; (2) high-grade trochlear dysplasia (grades B, C or D of Dejour’s classification [29]); (3) concomitant ligament reconstruction (cruciate ligament or collateral ligament); (4) open growth plate; (5) valgus or torsional deformities of the lower extremities; (6) fracture of distal femur or proximal tibia; (7) patella alta with Caton–Deschamps index greater than 1.2; (8) generalized or localized joint laxity; (9) rheumatoid arthritis or osteonecrosis; (10) incomplete medical records or imaging data and refusal to take part in this study. Patients with criteria (2), (5) and (7) had to receive both MPFLr and an additional bony procedure, including trochleoplasty, derotational distal femoral osteotomy and distal TTT, and thus were not included.

**Fig. 1:**
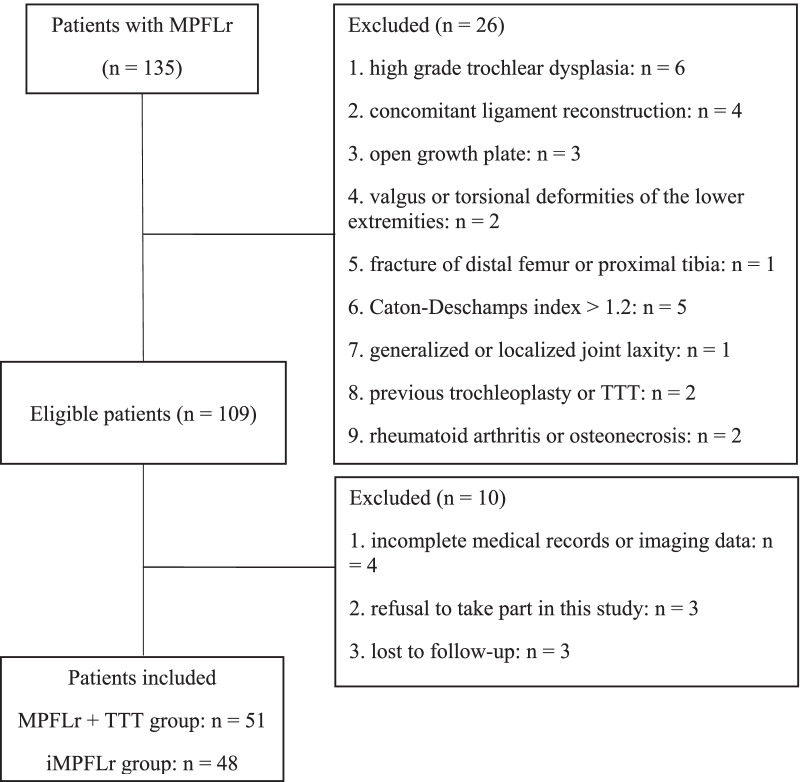
Flowchart of the patients in the MPFLr + TTT and iMPFLr groups. *iMPFLr* Isolated medial patellofemoral ligament reconstruction, *TTT* Tibial tubercle transfer

Based on these criteria, 102 MPFLr procedures in 102 patients, with a mean follow-up of 25.8 ± 7.6 months (range 12.5–33.2 months) were included. All these patients failed a conservative treatment and were followed up for at least one year after surgery. Three patients were lost to follow-up. A simultaneous medial TTT was performed if preoperative TT–TG distance was greater than 20 mm. Patients were divided into two groups according to whether they had undergone TTT or not. The MPFLr + TTT group consisted of 51 patients (51 knees) who underwent MPFLr combined with TTT. The iMPFLr group comprised of 48 patients (48 knees) who underwent iMPFLr (Fig. 1).

Preoperative radiographic examination consisted of anteroposterior, lateral and axial radiographs and axial CT scans in all patients. Caton–Deschamps index was measured on the lateral radiograph, which was defined as the ratio of the shortest distance from the lowest point of patellar articular surface to the anterior upper corner of tibial plateau contour to the length of patellar articular surface [30]. A Caton–Deschamps index greater than 1.2 was considered as patella alta. Trochlear dysplasia was evaluated on the lateral radiograph. According to Dejour’s classification, types B, C and D were regarded as high-grade trochlear dysplasia [29]. The TT–TG distance was measured on two overlapped axial CT images, including the deepest point of the trochlear groove and approximately proximal one-third of the TT. Two lines were drawn from the deepest point of the trochlear groove and the center of the TT, respectively, pendicular to the posterior condylar line. The distance between these two lines was measured as TT–TG distance [8].

### Surgical procedures

All surgical procedures were performed by the same experienced surgeon. First, a routine arthroscopy was performed through anterolateral and anteromedial portals to carefully evaluate the cartilage of patella and femoral trochlea. The cartilage injuries were graded according to the Outerbridge classification. Then the patellar tracking was checked within the range of motion of the knee.

The TTT was performed first and followed by the MPFLr. The amount of the TT medialization was evaluated by the preoperative CT measurement of TT–TG distance. TT–TG distance was restored to less than 20 mm, but care was taken not to overcorrect it. The TT was fixed with lag screws after obtaining a satisfactory patellar tracking. The MPFLr was completed using ipsilateral semitendinosus autograft. The graft was prepared in a “Y” shape to have a double bundle anatomical MPFLr. The folded end was whipstitched about 2.5 cm from the free end with Ethicon non-absorbable suture. Positions of the femoral and patellar tunnels were based on the native MPFL anatomy. The absorbable suture passed through the folded end and entered into the femoral tunnel, which was positioned between the medial femoral epicondyle and the adductor tubercle. Two bony grooves were made in the medial edge of the patella, which were located in the upper corner and the center of the patella, respectively. Three ends of the graft were fixed with absorbable screws.

At the end of the procedure, patellar tracking, range of motion (ROM) and lateral displacement of patella were examined. If the tightness of lateral patellar retinaculum produced tension in the MPFLr or restricted the patella from returning to the normal tracking, further release of the lateral retinaculum was performed.

### Postoperative rehabilitation

All patients followed the standard postoperative rehabilitation program. After procedure, the patient was placed in a brace to protect the knee. In the first 2 weeks postoperatively, passive ROM up to 60° and partial weight-bearing exercise with the knee held in extension were permitted. In the next 4 weeks, ROM was gradually increased to 90°. After 6 weeks, ROM was increased with no restriction and full weight-bearing exercise was allowed. The quadriceps femoris strength training and straight leg raising exercises were encouraged to strengthen the muscle early following the surgery. Patients who achieved full ROM and normal muscle strength and stability were allowed to return to normal sports activities at 6 months. Strenuous high-risk exercise required a longer rehabilitation period according to individual situation.

### Radiological evaluation

All the radiological evaluation was performed before and after surgery, including patellar tilt angle (PTA) and bisect offset (BO), which were measured on two overlapped axial CT images, including the widest patellar axis and the deepest point of trochlear groove, respectively. CT was performed in a standardized manner, with the patient in supine position, and the knee in full extension.

**Fig. 2:**
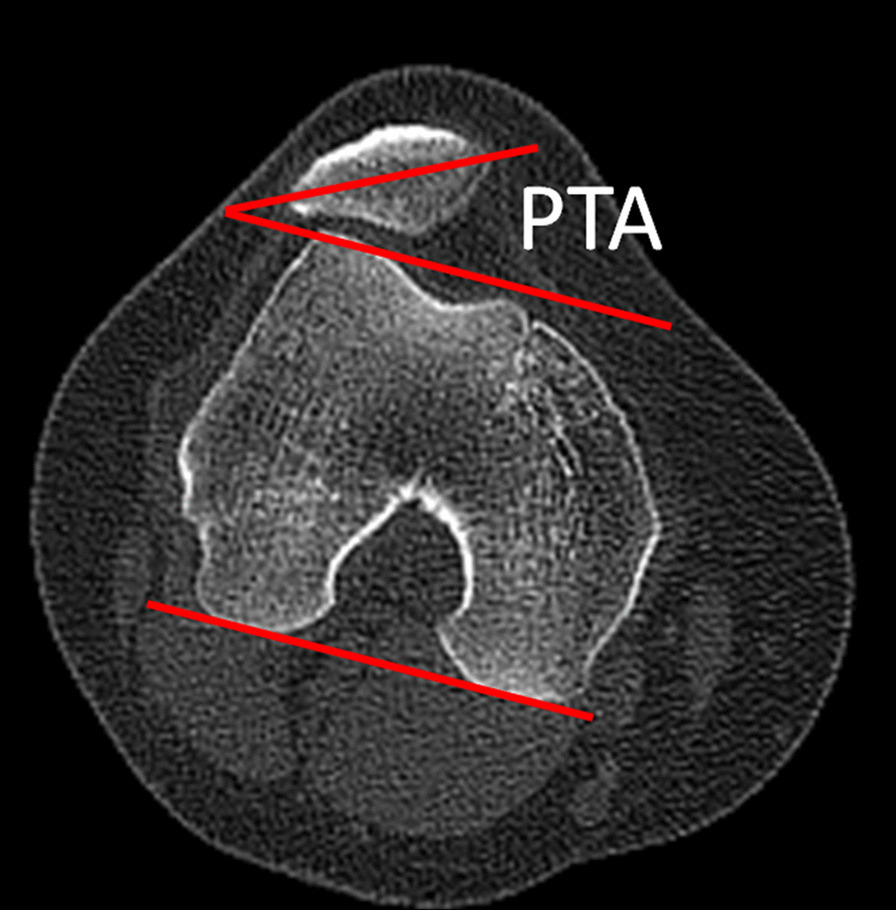
The measurement of the patellar tilt angle (PTA), defined as the angle between the widest patellar axis and the posterior condylar line

**Fig. 3:**
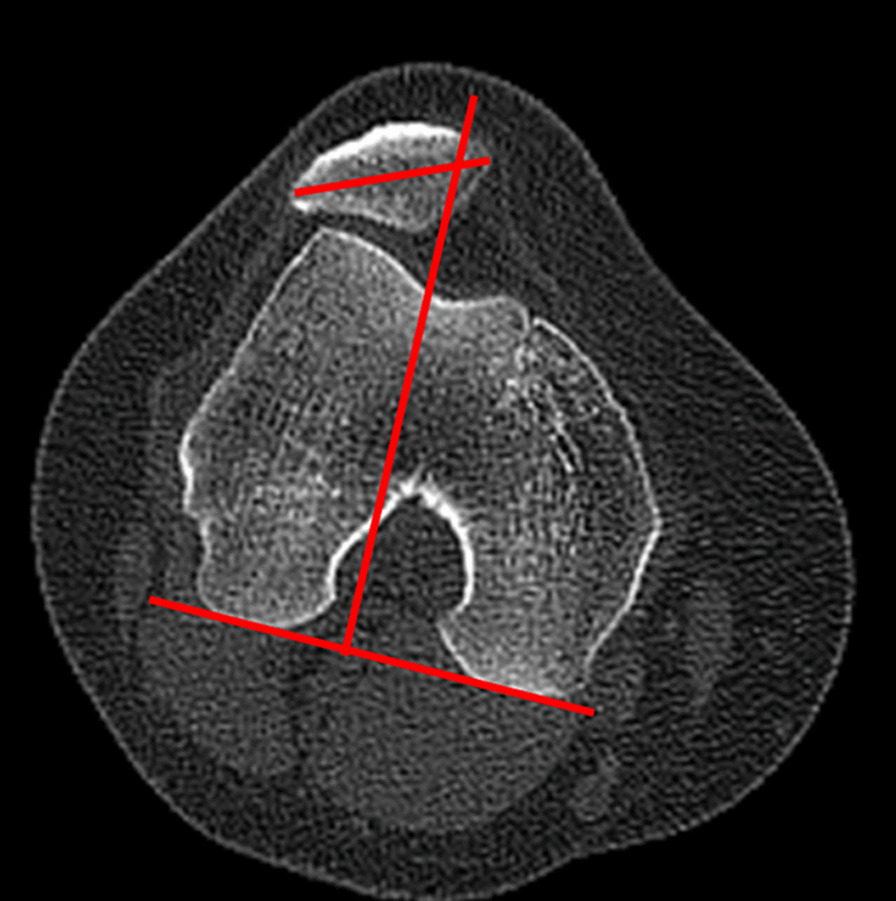
The measurement of the bisect offset (BO), defined as the portion of the width of the patella lateral to the deepest point of the trochlear groove

The PTA was defined as the angle between the widest patellar axis and the posterior condylar line (Fig. 2). The axial slice with the widest patella was determined, and the line connecting the medial and lateral edges of the patella was the widest patellar axis. The posterior condylar line was defined as the line passing through the most posterior points of the medial and lateral femoral condyles on the axial slice showing the posterior condyles with the Roman arch [31]. Positive value represented lateral patellar tilt. The BO indicated the lateral displacement of patella relative to the trochlear groove. A line was drawn through the deepest point of the trochlear groove and perpendicular to the posterior condylar line. The BO was measured as the portion of the width of the patella lateral to the deepest point of the trochlear groove (Fig. 3) [32]. A higher percentage represented a more lateral displacement of patella.

### Clinical evaluation

All the clinical evaluation was performed before and after surgery, including the QoL, functional outcomes, physical examination and redislocation rate. The QoL was evaluated using EQ-5D-5L, which is based on five dimensions [26]. Each dimension has five response categories: no problems, some problems, moderate problems, severe problems and extreme problems [27]. Patients also assessed their overall health on the vertical EQ-VAS with the range of 0–100 [26]. Functional outcomes included Kujala score, Lysholm score and Tegner activity score [33–35]. Kujala or Lysholm improvement was defined as the average change measured as the difference between the postoperative and preoperative Kujala or Lysholm scores. Functional failure was based on positive apprehension sign, recurrent patellar subluxation or dislocation, subjective instability and complications. The physical examination consisted of patellar apprehension test and the ROM. The patellar apprehension test was performed with the patient in the supine position and the quadriceps femoris relaxed at 30° of knee flexion. The experienced examiner put his thumb on the medial edge of the patella to push the patella laterally. The apprehension during the passive patellar glide was considered as a positive apprehension sign. The ROM was assessed with the patient in the supine position without weight bearing. An experienced examiner used a standard handheld goniometer to measure the maximum active flexion and extension angles. The redislocation rates were also recorded.

### Statistical analysis

Descriptive statistics were reported as mean ± standard deviation (SD) for continuous variables. Statistical comparisons were performed using SPSS Statistics software (version 21, SPSS Inc., Chicago, IL, USA) and *p* < 0.05 was considered to be significant. Shapiro–Wilk test was used to examine normality of the data. The paired and independent *t* tests were used for the data following a normal distribution. Otherwise, the Wilcoxon and Mann–Whitney U tests were used to analyze the differences. Categorical variables were compared by chi-square or Fisher’s exact test. The sample size was estimated using G*Power 3 (Heinrich Heine Universität Düsseldorf, Germany), based on the EQ-5D data collected in this study from included patients. A priori analysis indicated that the minimum required sample size with an effect size of 0.6, α of 0.05 and a power of 0.80 was 90. To evaluate intra-observer and inter-observer reliability, intra-class correlation (ICC) values were calculated. The measurements were either repeated by one researcher at the interval of two weeks or independently performed by two different researchers.

## Results

### Demographic data

The MPFLr + TTT group included 51 patients (51 knees), with an average age of 20.67 ± 5.72 years and an average body mass index (BMI) of 24.15 ± 4.60. This group included 22 males and 29 females. The iMPFLr group included 48 patients (48 knees), with an average age of 20.65 ± 7.57 years and an average BMI of 23.28 ± 3.66. This group included 18 males and 30 females. The mean TT–TG distance decreased from 22.18 ± 4.25 mm (range 21.25–29.30 mm) before surgery to 15.05 ± 4.18 mm (range 11.37–18.54 mm) after surgery in the MPFLr + TTT group, showing significant improvements (*p* < 0.001). The mean preoperative TT–TG distance in the iMPFLr group was 16.49 ± 2.59 mm (range 13.47–19.14 mm), with no significant difference between preoperative and postoperative values (*p* = 0.640). The groups were comparable for gender, age and BMI. Demographic data and knee characteristics are shown in Table 1.

**Table 1:** Demographic data and knee characteristics in the MPFLr + TTT group and iMPFLr group

	MPFLr + TTT group	iMPFLr group	*p* value
Number of patients	51	48	–
Number of knees	51	48	–
Gender (Male/female)	22/29	18/30	0.568
Side (left/right)	32/19	23/25	0.138
Age (year)	20.67 ± 5.72	20.65 ± 7.57	0.993
Body mass index	24.15 ± 4.60	23.28 ± 3.66	0.530
Follow-up (month)	24.9 ± 10.8	26.0 ± 11.3	0.318
Preoperative TT–TG distance (mm)	22.18 ± 4.25	16.49 ± 2.59	< 0.001

Data are reported as mean ± SD unless otherwise indicated. *iMPFLr* isolated medial patellofemoral ligament reconstruction

*TTT* Tibial tubercle transfer, *TT–TG* Tibial tubercle–trochlear groove

### QoL

**Fig. 4:**
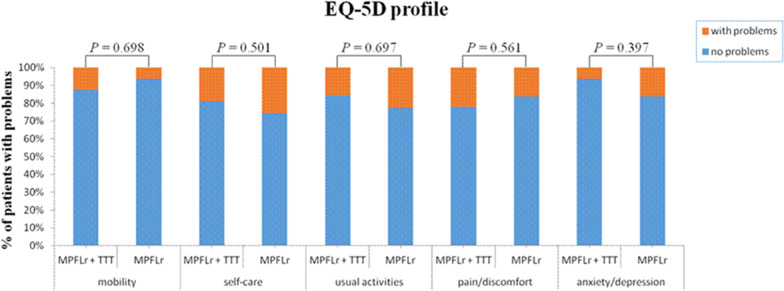
Percentage of patients who had problems in the five dimensions of the EQ-5D at the final follow-up in the MPFLr + TTT and iMPFLr groups. *iMPFLr* Isolated medial patellofemoral ligament reconstruction, *TTT* Tibial tubercle transfer

The EQ-5D index and EQ-5D VAS were significantly improved between baseline and the final follow-up in the two groups (*p* < 0.001 and *p* < 0.001, respectively). There was no significant difference in the EQ-5D index at baseline and the final follow-up between the two groups (*p* = 0.474 and *p* = 0.502, respectively). There was no significant difference in the EQ-5D VAS at baseline and the final follow-up between the two groups (*p* = 0.301 and *p* = 0.142, respectively) (Table 2). Five dimensions of EQ-5D at the final follow-up were also compared between the groups, but no significant difference was found, although percentages of people with problems of mobility and pain/discomfort were higher in the MPFLr + TTT group (Fig. 4). Female patients had lower EQ-5D index and EQ-5D VAS compared with male patients in both groups at the final follow-up, but there was only a significant difference in the EQ-5D VAS (Table 3).

**Table 2:** Preoperative and postoperative EQ-5D index and EQ-5D VAS in the MPFLr + TTT group and iMPFLr group

	MPFLr + TTT group (*n* = 51)	iMPFLr group (*n* = 48)	*p* value
*EQ-5D index*			
Baseline	0.63 ± 0.09	0.65 ± 0.09	0.474
Final follow-up	0.96 ± 0.04	0.95 ± 0.04	0.502
*EQ-5D VAS*			
Baseline	70.33 ± 5.96	68.18 ± 6.71	0.301
Final follow-up	89.29 ± 6.64	91.88 ± 3.87	0.142

Data are reported as mean ± SD

*iMPFLr* Isolated medial patellofemoral ligament reconstruction, *TTT* Tibial tubercle transfer

**Table 3:** Postoperative EQ-5D index and EQ-5D VAS in the MPFLr + TTT group and iMPFLr group according to gender

	MPFLr + TTT group (*n* = 51)		iMPFLr group (*n* = 48)	
	EQ-5D index	EQ-5D VAS	EQ-5D index	EQ-5D VAS
Gender				
Female	0.95 ± 0.04	86.67 ± 5.94	0.96 ± 0.04	90.86 ± 3.46
Male	0.98 ± 0.05	95.83 ± 2.40	0.99 ± 0.03	96.67 ± 0.58
*p* value	0.135	0.002	0.353	0.013

Data are reported as mean ± SD

*iMPFLr* Isolated medial patellofemoral ligament reconstruction, *TTT* Tibial tubercle transfer

### Functional outcomes and clinical results

Between baseline and the final follow-up, significant improvements for Kujala, Lysholm and Tegner activity scores were observed in both groups (*p* < 0.001), with no statistic difference between the two groups (Table 4). Although Kujala improvement was higher in the MPFLr + TTT group than iMPFLr group, there was no significant difference (*p* = 0.534). There was also no significant difference in Lysholm and Tegner activity improvements between the two groups (*p* = 0.813 and *p* = 0.723, respectively) (Table 4). Apprehension sign were negative in all patients.

**Table 4:** Preoperative and postoperative functional outcomes and clinical results in the MPFLr + TTT group and iMPFLr group

	MPFLr + TTT group (*n* = 51)	iMPFLr group (*n* = 48)	*p* value
*Kujala score*			
Baseline	60.90 ± 9.20	62.24 ± 8.13	0.644
Final follow-up	86.10 ± 5.02	85.18 ± 5.79	0.604
Kujala improvement	25.19 ± 12.27	22.94 ± 9.13	0.534
*Lysholm score*			
Baseline	48.76 ± 6.16	49.89 ± 4.80	0.925
Final follow-up	88.38 ± 2.94	87.65 ± 3.28	0.472
Lysholm improvement	38.62 ± 7.63	38.06 ± 6.62	0.813
*Tegner activity score*			
Baseline	3.14 ± 0.95	3.53 ± 0.94	0.223
Final follow-up	5.14 ± 1.15	5.35 ± 1.17	0.583
Tegner improvement	2.00 ± 1.61	1.82 ± 1.38	0.723
*Range of motion (°)*			
Flexion	130.6 ± 4.9	133.4 ± 5.2	0.098
Extension	1.8 ± 1.2	1.3 ± 0.9	0.160
Time to return to pre-injury activity level (month)	4.6 ± 2.2	4.4 ± 1.9	0.341

Data are reported as mean ± SD

*iMPFLr* Isolated medial patellofemoral ligament reconstruction, *TTT* Tibial tubercle transfer

There was no significant difference in the postoperative ROM between the two groups (*p* = 0.160 and *p* = 0.098, respectively). The average extension angle of ROM was 1.8° ± 1.2° and the average flexion was 130.6° ± 4.9° in the MPFLr + TTT group. The average extension angle of ROM was 1.3° ± 0.9° and the average flexion was 133.4° ± 5.2° in the iMPFLr group (Table 4). No extension and flexion deficit of 5° or more was reported in ROM in both groups at the final follow-up. Two patients (3.9%) in MPFLr + TTT group and three in iMPFLr group (6.3%) reported redislocation without apprehension, but only two patients sought medical treatment. They were treated conservatively because of no surgical indications.

Forty-four patients (86.3%) in MPFLr + TTT group and 42 (87.5%) in iMPFLr group were able to return to their pre-injury activity level at a mean of 4.6 ± 2.2 months and 4.4 ± 1.9 months, respectively (*p* = 0.341). A total of 90.2% (*n* = 46) of patients in MPFLr + TTT group and 93.8% (*n* = 45) in iMPFLr group were satisfied with the clinical outcomes, and they would like to recommend their surgeries to others.

### Radiological results

The PTA and BO were improved significantly between baseline and the final follow-up in the two groups (*p* < 0.001 and *p* < 0.001, respectively). There was no significant difference in the PTA and BO between the two groups at baseline and final follow-up, although PTA in the iMPFLr group was lower than MPFLr + TTT group (*p* = 0.051 and *p* = 0.381, respectively) (Table 5). Five patients in the MPFLr + TTT group and seven patients in the iMPFLr group still had a pathological patellar tilt postoperatively. All of these patients showed preoperative pathological patellar tilt greater than 30°. The intra-observer and inter-observer reliability of radiological measurements was good to excellent, with all ICCs greater than 0.8.

**Table 5:** Radiological results in the MPFLr + TTT group and iMPFLr group

	MPFLr + TTT group (*n* = 51)	iMPFLr group (*n* = 48)	*p* value
*Patellar tilt angle*			
Baseline	33.87 ± 8.71	29.42 ± 4.50	0.051
Final follow-up	16.13 ± 8.25	13.63 ± 9.14	0.381
*Bisect offset*			
Baseline	0.95 ± 0.10	0.95 ± 0.07	0.965
Final follow-up	0.70 ± 0.15	0.66 ± 0.08	0.333

Data are reported as mean ± SD

*iMPFLr* Isolated medial patellofemoral ligament reconstruction, *TTT* Tibial tubercle transfer

## Discussion

In this retrospective comparative study, clinical, functional and radiological results between 51 patients (51 knees) who underwent MPFLr + TTT and 48 patients (48 knees) who underwent iMPFLr for RPD were compared. The most important findings of this study were the significant improvements in the QoL following MPFLr + TTT and iMPFLr, with no significant difference in the EQ-5D index and EQ-5D VAS at baseline and the final follow-up between the two groups. Female patients had lower EQ-5D index and EQ-5D VAS compared with male patients in both groups. In addition, no significant difference was found in the functional outcomes, physical examinations, redislocation rates and radiological results between the two surgeries.

Decision making is complex, and clear treatment guidelines are still indeterminate for patients with RPD. The MPFLr, which was the standard treatment for RPD based on the consensus of International Patellofemoral Study Group, has been widely performed to restore the length and stiffness of the medial soft tissue [36]. However, patellar dislocation is a multifactorial condition, which depends on bony variables besides ligament laxity, including lateralization of TT [5, 6]. In patients with abnormally lateralized TT, iMPFLr is not sufficient to compensate for the lack of bony restraint and to restore patellofemoral pressure and dynamics [37]. Wagner et al. reported that patients with an increased TT–TG distance who underwent iMPFLr had a lower Kujala score, suggesting that medializing the TT when the TT–TG distance was greater than 20 mm was recommended [25]. Therefore, TTT may be necessary in these patients to decrease the pressure of lateral patella and trochlea. The purpose of TTT is to restore the relationship between femoral trochlea and TT, which can realign the extensor mechanism and increase patellofemoral stability [11, 37]. A systematic review demonstrated significant improvements in the overall clinical results, with low functional failure rates and reoperation rates following MPFLr + TTT [38]. However, no consensus has been reached regarding the threshold of the TT–TG distance as an indication for TTT. We took a TT–TG distance greater than 20 mm as the cutoff value for TTT in this study, which was Dejour et al. considered as abnormal [29].

Numerous studies reported significant improved QoL after iMPFLr using different methods, including EQ-5D-3L, Knee Injury and Osteoarthritis Outcome Score (KOOS), Pediatric International Knee Documentation Committee Form (Pedi-IKDC), Banff Patella Instrumentation and Short Form 12 (SF-12), but none of them mentioned psychometric effects [14, 36, 39, 40]. Bouras et al. used EQ-5D-3L questionnaire to evaluate the Qol after iMPFLr and noted that EQ-5D index and EQ-5D VAS significantly increased at last follow-up [14]. Biesert et al. also reported overall good health with EQ-5D-3L [41]. Erickson et al. found statistically significant improvement in mean KOOS-QoL [39]. However, to our knowledge no study has specifically investigated QoL and health state preference value following MPFLr + TTT for RPD with EQ-5D-5L. In this study, the EQ-5D index and EQ-5D VAS were significantly improved in the two groups. However, there was no significant difference in the EQ-5D index and EQ-5D VAS at baseline and the final follow-up between the two groups. Five dimensions of EQ-5D at the final follow-up were also compared between the groups, but no significant difference was found, although percentages of people with problems of mobility and pain/discomfort were higher in the MPFLr + TTT group. The small incision and decreased pain caused by the hardware, which were beneficial to the postoperative rehabilitation and activities, could account for the slightly higher QoL in the iMPFLr group. But this does not affect the significant improvement of the QoL following MPFLr + TTT. Our results were slightly lower than previous reported scores using EQ-5D-3L, which could be the results of reduced ceiling effect of EQ-5D-5L and a small sample size of this study [14, 41].

The reasons for improved QoL were multifactorial. One possible reason was the standard postoperative rehabilitation protocol adopted in this study, including rapid postoperative activities and quadriceps femoris strength training, which prevented muscle atrophy caused by immobilization. Another reason was that MPFLr and TTT were able to reestablish the normal anatomy of the knee, promote normal joint reaction forces and restore patellofemoral stability, which contributed to the relief of pain and related symptoms that affected general health conditions, including mental health [40]. Therefore, postoperative improvements in clinical evaluation, including functional outcomes, and radiological evaluation lead to the improvement of Qol. This could also explain the high postoperative satisfaction of patients.

The TT–TG distance is used as a decision-making parameter in TTT surgery. However, validity of the TT–TG distance has been brought into question. The TT–TG distance has a significant association with knee rotation, but it does not indicate the patellofemoral alignment and provide accurate information about the congruence between the patella and trochlear [42, 43]. Besides, TT–TG distance does not determine the location of the patellofemoral malformation [44]. Chen et al. reported that TT–TG distance was influenced more by knee rotation and trochlear groove medialization, instead of TT lateralization. Therefore, a high TT–TG distance might not be an appropriate indication for surgical planning [45]. This could also be the reason why our results showed that there was no significant difference in QoL between the two groups.

We found worse QoL assessed by the EQ-5D index and EQ-5D VAS in females versus males in both groups, but there was only a significant difference in the EQ-5D VAS. Bouras et al. also found EQ-5D index and EQ-5D VAS of female patients at baseline and final follow-up were lower than males following iMPFLr, but they used EQ-5D-3L and did not include iMPFLr + TTT group [14]. Other studies demonstrated poorer functional outcomes for females after iMPFLr + TTT. Allen et al. reported female gender was the risk factor for the lower scores of the International Knee Documentation Committee (IKDC) and Kujala score following MPFLr + TTT [46]. Compared with males, the reoperation rate of females was higher following MPFLr + TTT [20]. However, Watanabe et al. studied the efficacy of MPFLr with or without TTT and found no effect of gender on the results [12]. Migliorini et al. also reported that gender had no influence on surgical outcomes following iMPFLr [47]. One possible reason for lower QoL in females could be the higher probability of dysplasia and more joint laxity than males [12, 48]. Also, pain levels are usually severer in females following cartilage injury and the incidence of patellofemoral pain syndrome is also relatively high in females [46, 48]. This study supports that gender is an important risk factor of patellar instability.

Functional outcomes in this study, including Kujala, Lysholm and Tegner activity scores, were significantly improved in both groups with MPFLr + TTT or iMPFLr. However, no significant difference was found between the two groups. These findings are consistent with previously reported results. Schottle et al. showed no significant difference in Kujala score and subjective assessment between patients who underwent MPFLr with or without TTT [49]. Watanabe et al. reported no significant difference in the Lysholm score, which improved from 70 to 92 in the iMPFLr group and from 72 to 90 in the MPFLr + TTT group [12]. Neri et al. showed no difference in Kujala score [13]. Kim et al. also found no significant difference in Kujala and Tegner activity scores between the two groups [36]. However, Franciozi et al. reported Kujala and Lysholm improvements from baseline, favoring MPFLr + TTT over iMPFLr. But this TTT included anteriorization, which provided some biomechanical advantages for improving patellar tracking [23].

This study reported low redislocation rate following MPFLr + TTT. Two patients in the MPFLr + TTT group and three in the iMPFLr group reported redislocation without apprehension. This corresponds with findings of other studies. Kim et al. reported no difference in the functional failure and complications, with two subjective instability and one repeat dislocation in MPFLr + TTT group, and two subjective instability in iMPFLr group [36]. Allen et al. reported one dislocation and one subluxation after MPFLr + TTT [46]. Cossey et al. showed that no recurrence of subluxation or dislocation occurred and objective stability was excellent after MPFLr with TTT [50]. Although the iMPFLr provides restraint which prevents against redislocation, an uncorrected TT–TG distance could still cause episodes of instability or dislocation during activity.

CT scans demonstrated an improvement in the PTA and BO in both MPFLr + TTT group and iMPFLr group, but no significant difference was observed in two groups. Similar findings were also reported in other studies. Kim et al. reported no significant difference between the two groups in PTA with significant improvement [36]. Neri et al. also reported similar results regarding PTA [13]. A biomechanical study showed that MPFLr significantly reduced BO, especially at low flexion angles [51]. PTA and BO were improved, because of the dorsal tension of the medial edge of patella by the graft and the medial tension on the patella caused by medialization of TT [48]. However, overcorrection of patellar tilt due to the overtension of the graft could increase the medial patellofemoral contact pressure and result in the damage of the medial patellofemoral cartilage, thus developing into osteoarthritis [13]. Whether these results can reflect patellar tracking and improve patellofemoral pressure remains to be studied, which can reduce long-term cartilage wear and osteoarthritis.

This study has some limitations. First, the sample size of patients was relatively small, and the follow-up time was not long enough to evaluate more significant clinical and radiological results. Second, TT–TG distance was measured by CT images with knee extension. Therefore, the results could be different from those obtained from CT or MR images with different knee angles. Third, because different patients had different evaluation time, the results could vary from time to time. Fourth, the additional incision of TTT procedure and the existence of hardware made it difficult to use blind methods in clinical and radiological evaluation, which could lead to measurement bias. A prospective controlled study with a large sample and a long follow-up is required to further confirm our findings.

## Conclusion

In the current study, both MPFLr + TTT and iMPFLr groups obtained similar and satisfactory improvements in the QoL, clinical results and radiological outcomes. There was no significant difference in the EQ-5D index and EQ-5D VAS at baseline and the final follow-up between the two groups, indicating that MPFLr combined with TTT is a safe and effective procedure, which can significantly improve the QoL for patients with RPD in cases of pathologically lateralized TT. However, female patients obtained lower QoL than males.

## Data Availability

The datasets used and analyzed during the current study are available from the corresponding author on reasonable request.

## Abbreviations

MPFLr: Medial patellofemoral ligament reconstruction
TTT: Tibial tubercle transfer
QoL: Quality of life
RPD: Recurrent patellar dislocation
TT–TG: Tibial tubercle–trochlear groove
EQ-5D-5L: Five-level EuroQol five-dimensional questionnaire
VAS: Visual analogue scale
CT: Computed tomography
MR: Magnetic resonance
ROM: Range of motion
PTA: Patellar tilt angle
BO: Bisect offset
SD: Standard deviation
ICC: Intra-class correlation
KOOS: Knee Injury and Osteoarthritis Outcome Score
IKDC: International Knee Documentation Committee
SF: Short form

## References

[1] Wang XL, Peng C, Tu YW, Liu YP, Zhang W, Zhang Y (2021). Effects of lateral patellar retinaculum release for recurrent patella dislocation: a prospective study. Int J Gen Med.

[2] Wilkens OE, Hannink G, van de Groes S (2020). Recurrent patellofemoral instability rates after MPFL reconstruction techniques are in the range of instability rates after other soft tissue realignment techniques. Knee Surg Sports Traumatol Arthrosc.

[3] Sanders TL, Pareek A, Hewett TE, Stuart MJ, Dahm DL, Krych AJ (2018). Incidence of first-time lateral patellar dislocation: a 21-year population-based study. Sports Health.

[4] Takagi S, Sato T, Watanabe S, Tanifuji Q, Mochizuki T, Omori G (2018). Alignment in the transverse plane, but not sagittal or coronal plane, affects the risk of recurrent patella dislocation. Knee Surg Sports Traumatol Arthrosc.

[5] Colvin AC, West RV (2008). Patellar instability. J Bone Joint Surg Am.

[6] Williams AA, Elias JJ, Tanaka MJ, Thawait GK, Demehri S, Carrino JA (2016). The relationship between tibial tuberosity-trochlear groove distance and abnormal patellar tracking in patients with unilateral patellar instability. Arthroscopy.

[7] Zhang Z, Cao Y, Song G, Li Y, Zheng T, Zhang H (2021). Derotational femoral osteotomy for treating recurrent patellar dislocation in the presence of increased femoral anteversion: a systematic review. Orthop J Sports Med.

[8] Matsushita T, Kuroda R, Oka S, Matsumoto T, Takayama K, Kurosaka M (2014). Clinical outcomes of medial patellofemoral ligament reconstruction in patients with an increased tibial tuberosity-trochlear groove distance. Knee Surg Sports Traumatol Arthrosc.

[9] Marín Fermín T, Migliorini F, Kalifis G, Zikria BA, D’Hooghe P, Al-Khelaifi K (2022). Hardware-free MPFL reconstruction in patients with recurrent patellofemoral instability is safe and effective. J Orthop Surg Res.

[10] Panni AS, Cerciello S, Maffulli N, Di Cesare M, Servien E, Neyret P (2011). Patellar shape can be a predisposing factor in patellar instability. Knee Surg Sports Traumatol Arthrosc.

[11] Vetrano M, Oliva F, Bisicchia S, Bossa M, De Carli A, Di Lorenzo L (2017). IS Mu. LT first-time patellar dislocation guidelines. Muscles Ligaments Tendons J.

[12] Watanabe T, Muneta T, Ikeda H, Tateishi T, Sekiya I (2008). Visual analog scale assessment after medial patellofemoral ligament reconstruction: with or without tibial tubercle transfer. J Orthop Sci.

[13] Neri T, Parker DA, Beach A, Gensac C, Boyer B, Farizon F (2019). Medial patellofemoral ligament reconstruction with or without tibial tubercle transfer is an effective treatment for patellofemoral instability. Knee Surg Sports Traumatol Arthrosc.

[14] Bouras T, Brown A, Gallacher P, Barnett A (2019). Isolated medial patellofemoral ligament reconstruction significantly improved quality of life in patients with recurrent patella dislocation. Knee Surg Sports Traumatol Arthrosc.

[15] Fulkerson JP (2018). Editorial commentary: medial patellofemoral ligament reconstruction alone works well when the patient has normal alignment, but don’t forget to move the tibial tubercle when necessary. Arthroscopy.

[16] Nelitz M, Williams RS, Lippacher S, Reichel H, Dornacher D (2014). Analysis of failure and clinical outcome after unsuccessful medial patellofemoral ligament reconstruction in young patients. Int Orthop.

[17] Stephen JM, Dodds AL, Lumpaopong P, Kader D, Williams A, Amis AA (2015). The ability of medial patellofemoral ligament reconstruction to correct patellar kinematics and contact mechanics in the presence of a lateralized tibial tubercle. Am J Sports Med.

[18] Frings J, Krause M, Wohlmuth P, Akoto R, Frosch KH (2018). Influence of patient-related factors on clinical outcome of tibial tubercle transfer combined with medial patellofemoral ligament reconstruction. Knee.

[19] Redler LH, Meyers KN, Brady JM, Dennis ER, Nguyen JT, Shubin Stein BE (2018). Anisometry of medial patellofemoral ligament reconstruction in the setting of increased tibial tubercle-trochlear groove distance and patella alta. Arthroscopy.

[20] Gorbaty JD, Varkey DT, Hong IS, Trofa DP, Odum SM, Piasecki DP (2022). Outcomes and reoperation rates after tibial tubercle transfer and medial patellofemoral ligament reconstruction: higher revision stabilization in patients with trochlear dysplasia and patella alta. Knee Surg Sports Traumatol Arthrosc.

[21] Burnham JM, Howard JS, Hayes CB, Lattermann C (2016). Medial patellofemoral ligament reconstruction with concomitant tibial tubercle transfer: A systematic review of outcomes and complications. Arthroscopy.

[22] Krych AJ, O’Malley MP, Johnson NR, Mohan R, Hewett TE, Stuart ML (2018). Functional testing and return to sport following stabilization surgery for recurrent lateral patellar instability in competitive athletes. Knee Surg Sports Traumatol Arthrosc.

[23] Franciozi CE, Ambra LF, Albertoni L, Debieux P, de Mello Granata Jr GS, Kubota MS (2019). Anteromedial tibial tubercle osteotomy improves results of medial patellofemoral ligament reconstruction for recurrent patellar instability in patients with tibial tuberosity-trochlear groove distance of 17 to 20 mm. Arthroscopy.

[24] Agarwalla A, Gowd AK, Liu JN, Puzzitiello RN, Yanke AB, Verma NN (2019). Concomitant medial patellofemoral ligament reconstruction and tibial tubercle osteotomy do not increase the incidence of 30-day complications: an analysis of the NSQIP database. Orthop J Sports Med.

[25] Wagner D, Pfalzer F, Hingelbaum S, Huth J, Mauch F, Bauer G (2013). The influence of risk factors on clinical outcomes following anatomical medial patellofemoral ligament (MPFL) reconstruction using the gracilis tendon. Knee Surg Sports Traumatol Arthrosc.

[26] Herdman M, Gudex C, Lloyd A, Janssen MF, Kind P, Parkin D (2011). Development and preliminary testing of the new five-level version of EQ-5D (EQ-5D-5L). Qual Life Res.

[27] Versteegh MM, Vermeulen KM, Evers SM, de Wit GA, Prenger R, Stolk AE (2016). Dutch tariff for the five-level version of EQ-5D. Value Health.

[28] Janssen MF, Birnie E, Haagsma JA, Bonsel GJ (2008). Comparing the standard EQ-5D three-level system with a five-level version. Value Health.

[29] Dejour H, Walch G, Nove-Josserand L, Guier C (1994). Factors of patellar instability: an anatomic radiographic study. Knee Surg Sports Traumatol Arthrosc.

[30] Caton J (1989). Method of measuring the height of the patella. Acta Orthop Belg.

[31] Kang H, Dong C, Tian G, Wang F (2019). A Computed tomography study of the association between increased patellar tilt angle and femoral anteversion in 30 patients with recurrent patellar dislocation. Med Sci Monit.

[32] Xue Z, Song GY, Liu X, Zhang H, Wu G, Qian Y (2018). Excessive lateral patellar translation on axial computed tomography indicates positive patellar J sign. Knee Surg Sports Traumatol Arthrosc.

[33] Kujala UM, Jaakkola LH, Koskinen SK, Taimela S, Hurme M, Nelimarkka O (1993). Scoring of patellofemoral disorders. Arthroscopy.

[34] Lysholm J, Gillquist J (1982). Evaluation of knee ligament surgery results with special emphasis on use of a scoring scale. Am J Sports Med.

[35] Tegner Y, Lysholm J (1985). Rating systems in the evaluation of knee ligament injuries. Clin Orthop Relat Res.

[36] Kim JM, Sim JA, Yang H, Kim YM, Wang JH, Seon JK (2021). Clinical comparison of medial patellofemoral ligament reconstruction with or without tibial tuberosity transfer for recurrent patellar instability. Am J Sports Med.

[37] Ahmad R, Calciu M, Jayasekera N, Schranz P, Mandalia V (2017). Combined medial patellofemoral ligament reconstruction and tibial tubercle transfer results at a follow-up of 2 years. J Knee Surg.

[38] Longo UG, Berton A, Salvatore G, Migliorini F, Ciuffreda M, Nazarian A (2016). Medial patellofemoral ligament reconstruction combined with bony procedures for patellar instability: current indications, outcomes, and complications. Arthroscopy.

[39] Erickson BJ, Nguyen J, Gasik K, Gruber S, Brady J, Shubin Stein BE (2019). Isolated medial patellofemoral ligament reconstruction for patellar instability regardless of tibial tubercle-trochlear groove distance and patellar height: outcomes at 1 and 2 years. Am J Sports Med.

[40] Herdea A, Pencea V, Lungu CN, Charkaoui A, Ulici A (2021). A prospective cohort study on quality of life among the pediatric population after surgery for recurrent patellar dislocation. Children (Basel).

[41] Biesert M, Johansson A, Kostogiannis I, Roberts D (2020). Self-reported and performance-based outcomes following medial patellofemoral ligament reconstruction indicate successful improvements in knee stability after surgery despite remaining limitations in knee function. Knee Surg Sports Traumatol Arthrosc.

[42] Caplan N, Lees D, Newby M, Ewen A, Jackson R, St ClairGibson A (2014). Is tibial tuberosity–trochlear groove distance an appropriate measure for the identification of knees with patellar instability. Knee Surg Sports Traumatol Arthrosc.

[43] Kaiser P, Loth F, Attal R, Kummann M, Schuster P, Riechelmann F (2020). Static patella tilt and axial engagement in knee extension are mainly influenced by knee torsion, the tibial tubercle-trochlear groove distance (TTTG), and trochlear dysplasia but not by femoral or tibial torsion. Knee Surg Sports Traumatol Arthrosc.

[44] Seitlinger G, Scheurecker G, Högler R, Labey L, Innocenti B, Hofmann S (2012). Tibial tubercle-posterior cruciate ligament distance: a new measurement to define the position of the tibial tubercle in patients with patellar dislocation. Am J Sports Med.

[45] Chen J, Wu C, Ye Z, Zhao J, Xie G (2022). Tibial tuberosity-trochlear groove distance and its components in patients with and without episodic patellar dislocation: a study of 781 knees. J Bone Joint Surg Am.

[46] Allen MM, Krych AJ, Johnson NR, Mohan R, Stuart MJ, Dahm DL (2018). Combined tibial tubercle osteotomy and medial patellofemoral ligament reconstruction for recurrent lateral patellar instability in patients with multiple anatomic risk factors. Arthroscopy.

[47] Migliorini F, Eschweiler J, Betsch M, Knobe M, Tingart M, Maffulli N (2022). Prognostic factors for isolated medial patellofemoral ligament reconstruction: a systematic review. Surgeon.

[48] Enderlein D, Nielsen T, Christiansen SE, Faunø P, Lind M (2014). Clinical outcome after reconstruction of the medial patellofemoral ligament in patients with recurrent patella instability. Knee Surg Sports Traumatol Arthrosc.

[49] Schöttle PB, Fucentese SF, Romero J (2005). Clinical and radiological outcome of medial patellofemoral ligament reconstruction with a semitendinosus autograft for patella instability. Knee Surg Sports Traumatol Arthrosc.

[50] Cossey AJ, Paterson R (2005). A new technique for reconstructing the medial patellofemoral ligament. Knee.

[51] Elias JJ, Tanaka MJ, Jones KC, Cosgarea AJ (2020). Tibial tuberosity anteriomedialization vs. medial patellofemoral ligament reconstruction for treatment of patellar instability related to malalignment: computational simulation. Clin Biomech (Bristol, Avon).

